# STING pathways and Sjögren’s syndrome: Exploration from mechanism to treatment

**DOI:** 10.3389/fimmu.2025.1649046

**Published:** 2025-09-22

**Authors:** Yuxiu Ka, Tingting Tan, Yihua Fan, Wei Liu, Aihua Wang, Wen Wang, Gesang Yuzhen, JingYi Zhang, Xiaoge Yao, Xueying Lin, Yuanhao Wu

**Affiliations:** ^1^ Department of Rheumatism and Immunity, First Teaching Hospital of Tianjin University of Traditional Chinese Medicine, Tianjin, China; ^2^ Department of Rheumatism and Immunity, National Clinical Research Center for Chinese Medicine Acupuncture and Moxibustion, Tianjin, China; ^3^ Department of Traditional Chinese Medicine Rheumatology, Shenzhen Nanshan People’s Hospital, Shenzhen, Guangdong, China; ^4^ Department of Rheumatism and Immunity, Hospital of Chengdu University of Traditional Chinese Medicine, Chengdu, Sichuan, China; ^5^ Department of Rheumatism and Immunity, Tianjin Third Central Hospital, Tianjin, China

**Keywords:** Sjögren’s syndrome, STING pathways, CGAS, STING inhibitor, review

## Abstract

Sjögren’s syndrome (SS) is an autoimmune disease characterized by abnormal lymphocyte proliferation and progressive exocrine gland dysfunction. The Stimulator of Interferon Genes (STING) pathways, as an important intracellular immune hub, overactivation can drive abnormally high expression of type I interferon and induce inflammatory cell infiltration, which is considered an important mechanism in the pathogenesis of SS. However, currently there is limited clinical evidence for direct activation of STING in human SS, and its tissue-specific regulatory mechanisms in target organs also need to be further elucidated. Based on this, STING pathway inhibitors have shown potential value in treating SS. This article systematically reviews the molecular mechanisms of the STING pathways in the pathogenesis of SS, explores its feasibility as a therapeutic target, and provides new evidence and ideas for precision treatment of SS.

## 1 Introduction

Sjögren’s syndrome (SS) is a chronic inflammatory autoimmune disorder predominantly characterized by lymphocytic infiltration into exocrine tissue and is common in women (1). Clinical manifestations include localized manifestations comprising oral dryness, ocular dryness, rampant dental caries, and adult parotitis, as well as various systemic manifestations such as fatigue, pain, and lymphoma (2–4). The exact etiology of the illness is unknown but may be related to genetic (e.g., HLA-DRB1, DQA1 genes), endocrine (e.g., sex hormone levels), and infection (e.g., hepatitis C virus, Epstein-Barr virus) factors (3, 5). The pathological mechanism involves the abnormal T and B lymphocyte activation, bringing about damage to glands and other organs (1, 3, 6).

The Stimulator of Interferon Genes (STING) (also referred to as TMEM173, MPYS, MITA, ERIS, etc.) is a key endoplasmic reticulum (ER) membrane protein in the natural immune reaction, which mediates the detection of cytoplasmic DNA, thereby activating type I interferon (IFN) synthesis and pro-inflammatory mediator secretion, and is vital in regulating antibacterial, antiviral, and antitumor immune responses *in vivo* (7–9). STING, consisting of four transmembrane helices (TM), a C-terminal tail (CTT), a cytoplasmic ligand-binding domain (LBD), and a short N-terminal cytoplasmic segment, is a transmembrane protein (10, 11). Among them, the short N-terminal cytoplasmic segment is involved in the interaction of other proteins and regulates the activity of STING. The TM domain resides within the ER membrane, anchoring STING to this compartment. After LBD is responsible for binding to the second messenger metabolite 2′,3′-cyclic-GMP-AMP (cGAMP), STING transitions from an inactive open conformation to an activated closed conformation. CTT is responsible for binding to TBK1, which in turn activates downstream signaling pathways (10, 12). Lately, the STING pathways have attracted widespread attention as a possible biomarker and intervention target in tumors, neurological diseases, kidneys, and others (13–17). The purpose of this paper focuses on exploring the function of the STING pathways in the progression of SS and offering new targets for its diagnosis and treatment.

## 2 Abnormal release of endogenous DNA

Cell-free DNA (cf-DNA) refers to DNA fragments from apoptotic or necrotic cells circulating in the blood. Research has shown that the levels of cf-DNA in the plasma and labial glands of pSS patients are significantly higher than those in the healthy control group and are associated with disease activity. Further analysis revealed a significant negative correlation between the levels of cf-DNA in the serum of pSS patients and the activity of deoxyribonuclease 1 (18). The serum levels of cf-DNA are higher in SS high-risk lymphoma patients and confirmed lymphoma patients, and there are a large number of extranuclear cells in PBMC and salivary gland (SG) tissues with DNA accumulation (19). Transmission electron microscopy showed alterations in mitochondrial structure in salivary gland epithelial cells (SGEC) of SS patients, such as mitochondrial matrix swelling, cristae loss and disorganization, mitochondrial membrane rupture, and myelin-like structures within the mitochondria, resulting in leakage of mitochondrial contents (20). Cytosolic mitochondrial DNA (mtDNA) release engages the cGAS-STING axis, inducing IFN-stimulated gene expression. mtDNA entering the cytosol triggers the cGAS-STING pathway and upregulates IFN-stimulated genes (13, 21). It was found that lactate levels in SS-affected SG were elevated, and the high-lactate environment caused mitochondrial damage and escape in SGEC. mtDNA activated the STING pathway and triggered the inflammatory response, promoted the proliferation of lymphocytes and inhibited apoptosis (22). The lactate scavenging agent DCA attenuates the glandular inflammation in NOD/Ltj mice (22). Patients with SS have been found to have an increased number of mtDNA copies (23). DNA from phagocytosed apoptotic cells leaks from the phagolysosomal compartment to trigger STING function (24). When the STING pathway is activated, it might initiate the pathogenesis of SS (25).

## 3 Activation of the STING pathway by nucleic acid receptors

**Figure 1:**
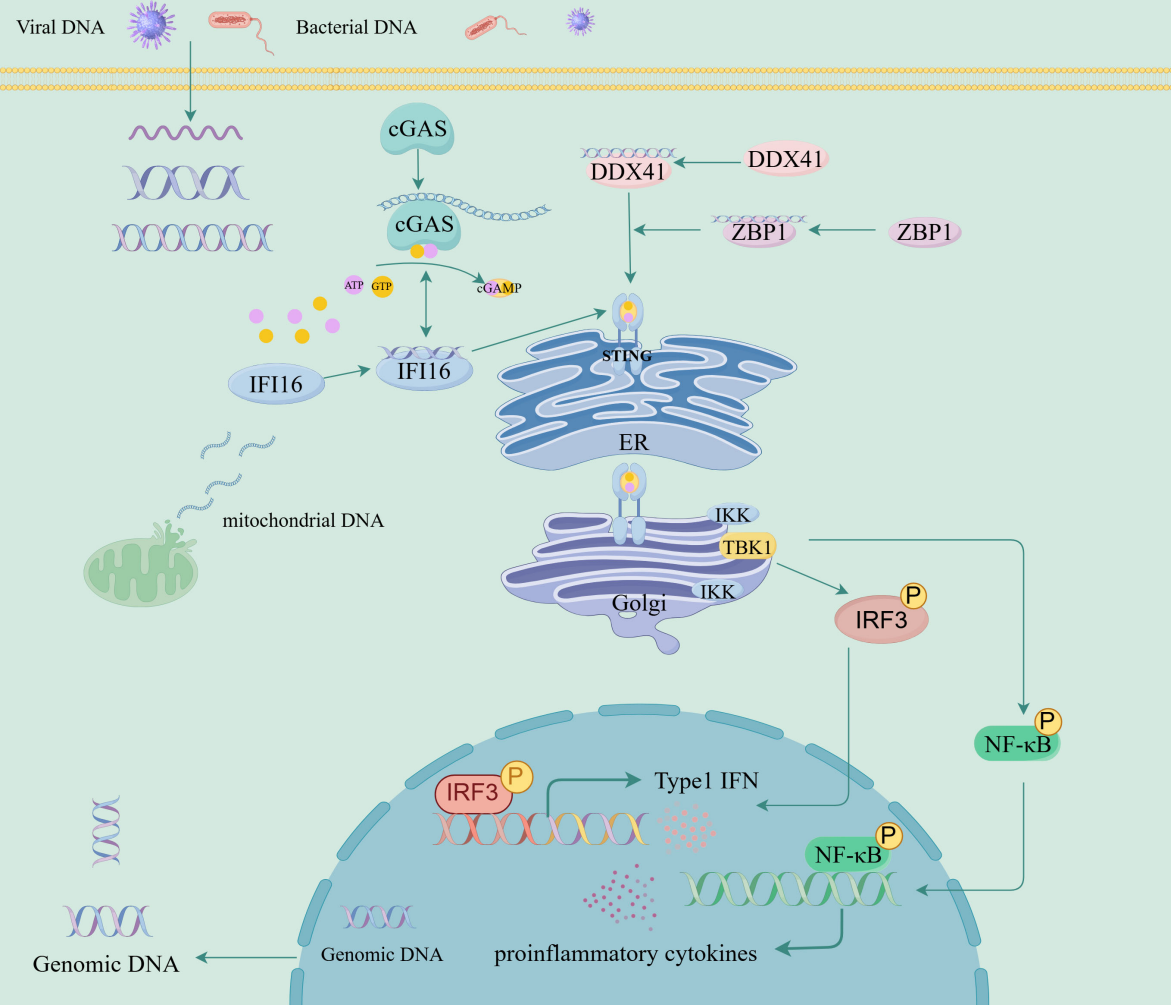
Interferon gene-stimulated receptor (STING) signaling pathway and its regulation.

The second messenger cGAMP synthase (cGAS), Interferon Gamma Inducible Protein 16 (IFI16), DEAD-box helicase 41 (DDX41), and DNA binding protein 1 (ZBP1) are all intracellular nucleic acid sensors that activate the STING pathway through different mechanisms, activate type I IFN, and produce pro-inflammatory cytokines (**Figure 1**, **Table 1**) (26). cGAS serves as a cytoplasmic DNA receptor that recognizes both exogenous (e.g., viral, bacterial) and endogenous DNAs (27). Binding of this protein to dsDNA drives the conversion of ATP and GTP into cGAMP (11, 28, 29). By binding to STING, cGAMP triggers its activation process, which subsequently causes STING to undergo a conformational transition, thereby promoting its ER-to-Golgi translocation, where it forms a multimeric structure and recruits and activates TBK1 and IKK, and induces NF-κB and IRF3 transcriptional activity, thereby stimulating the release of early inflammatory factors and the upregulation of type I IFN (7, 28, 30). IFI16 belongs to the PYHIN family (including Pyrin and HIN domains) and binds to dsDNA (such as viral genomic DNA or replication intermediates) in a sequence-independent manner through the HIN domain (31, 32). IFI16 interacts with cGAS in the DNA sensing process of human keratinocytes and macrophages (32, 33). IFI16 can also activate the cGAMP STING pathway by promoting STING phosphorylation and translocation (32). DDX41 is an RNA helicase of the DEAD box family and also a cytoplasmic DNA sensor (34). DDX41 directly binds to DNA and STING proteins through its DEADC domain, subsequently triggering the TBK1, NF-κB, and IRF3 signaling pathways (34–36). ZBP1 is a DNA sensor that induces type I IFN production and innate immune response (37). ZBP1 activates the STING pathway by binding its DNA domain to cytoplasmic DNA, driving the activation of IRF3 and promoting the transcription of type I IFN (38). During radiation-induced tumor cell necrosis, the ZBP1-MLKL necrotic apoptosis cascade induces cytoplasmic DNA accumulation, which in turn autonomously activates cGAS-STING signaling, forming a positive feedback loop between these two pathways and driving persistent inflammation (39).

**Table 1:** Core features of nucleic acid sensors in pSS.

Nucleic acid sensors	Identify substrates	Mechanism of activating the STING pathways	Expression in pSS	References
cGAS	Exogenous/endogenous dsDNA	Generate cGAMP, induce STING conformational changes and activate TBK1/IKK	Expressed in lip gland tissue and positively correlated with focal index	(11, 27–30, 41)
IFI16	Exogenous/endogenous dsDNA	Combining dsDNA with cGAS to promote STING phosphorylation/translocation and activate the cGAMP-STING pathway	High expression of pDC and monocytes	(31–33, 40)
DDX41	Exogenous/endogenous dsDNA	Directly binding to DNA and STING, triggering TBK1, NF-κB, and IRF3 pathways	High expression of pDC	(34–36, 40)
ZBP1	Exogenous/endogenous dsDNA (especially Z-DNA)	Combining cytoplasmic DNA, activating the STING pathway, driving IRF3 and type I IFN	There is expression in peripheral blood (specific expression changes not explicitly mentioned)	(37–39, 42)
PARP9	RNA	Not directly activating STING, enhancing type I IFN signaling through the PI3K/AKT3 pathway, or negatively regulate the expression of cGAS	Upregulation of expression in peripheral blood B cells and SG	(50–54)

Research has found that (40) compared to the healthy control group, monocytes in pSS patients exhibit highly reactive IFN-α production after STING stimulation. Compared with pSS with low IFN, pDCs with high IFN showed high expression of IFI16 and DDX41 and high expression of IFI16 in monocytes; the expression levels of cGAS and ZBP-1 are equal in monocytes and pDCs of pSS patients and healthy controls (40). However, some studies have found that there are a large number of cGAS-positive cells infiltrating the interstitial tissue of the labial gland tissue in pSS patients, and the expression level of cGAS in the labial gland tissue of pSS patients is correlated with the focal index (41). In the peripheral blood of pSS patients, the expression of ZBP1 is significantly upregulated, and this upregulation is closely related to the activation of the type I IFN signaling pathway (42). TRIM21 (also known as Ro52/SSA) is the main self-antigen of SS (43). pSS autoantibodies (anti-Ro/SSA autoantibodies) promote stimulation of the NF-κB cascade and the apoptotic pathway in human SGEC, resulting in expression of inflammatory cytokines and genes, exacerbating the inflammatory response (44, 45). In SS patients, overexpression of TRIM21 enhances the type I IFN pathway, leading to persistent inflammation (46). TRIM21 interacts with the DEADC domain of DDX41 through its PRYSPRY domain, mediating the K48 ubiquitination of DDX41, leading to degradation of DDX41 through the proteasome pathway and negatively regulating the innate immune response to intracellular dsDNA (47). TRIM21 also promotes IFI16 degradation through the ubiquitin proteasome pathway (48). TRIM21 and DDX41 are located together in the cytoplasm under resting conditions, and when stimulated by dsDNA, TRIM21 dissociates from DDX41 (49). In SS patients, there seems to be a contradictory phenomenon between the high expression of TRIM21 and its supposed inhibitory effect on type I IFN response, which may be due to functional defects of TRIM21 (such as structural abnormalities and post-translational modifications, leading to impaired ubiquitin ligase activity), significantly reducing its ability to degrade DDX41 and IFI16, resulting in abnormal accumulation of DDX41 and IFI16 in cells, activating the STING pathway, and ultimately leading to an overactive type I IFN response.

Identification of poly (ADP ribose) polymerase 9 (PARP9) can serve as a nonclassical RNA sensor that relies on the PI3K/AKT3 pathway to activate IRF3 and IRF7, thereby mediating the production of type I IFN (50). During Mycobacterium tuberculosis infection, PARP9 negatively regulates the expression of cGAS, cGAMP production, and downstream IFN-β production in macrophages (51). Research has found that the methylation level of the PARP9 gene is significantly reduced in peripheral blood B cells of pSS patients, leading to upregulation of its expression (51). This hypomethylation phenomenon suggests that PARP9 may participate in the pathogenesis of pSS by activating the type I IFN signaling pathway through epigenetic mechanisms (52). The study found that the level of PARP-9 in SG lesions of pSS patients increased with the increase of Chisholm score (53), indicating that the expression of PARP9 is correlated with the severity of SG lesions. Overexpression of PARP-9 and CXCL10 in SG infiltrating monocytes of the female NOD/LtJ mouse model, with PARP-9 upregulating IFIT-1 (mediated by STAT1 phosphorylation) to increase CXCL10 expression (53). The high expression of PARP9 can serve as a potential biomarker for pSS for early diagnosis and disease monitoring (54). The high expression of PARP9 is closely related to the activation of the type I IFN signaling pathway. Although there is currently no literature directly studying the interaction between PARP9 and the STING pathway in SS, PARP9 may indirectly affect the STING pathway by enhancing the type I IFN signaling pathway, thereby exacerbating the inflammatory response of pSS. Future research can further explore the potential of PARP9 as a biomarker and therapeutic target.

## 4 Histopathological effects of abnormal activation of the STING pathways

Research has shown that activation of the STING pathways can trigger abnormally high expression of type I IFN, which may be involved in the pathogenesis of pSS (**Table 2**) (55). The activation of the STING pathways is significantly manifested in SG, lacrimal glands, and lungs. The IFN-α/β pathway demonstrates pathological activation in SS (56). Interferon-stimulated genes (ISGs) are overexpressed in SG, peripheral blood mononuclear cells (PBMCs), ocular epithelial cells, SGEC, plasmacytoid dendritic cells, and B cells of SS patients (57–59). Studies have shown that nuclear translocation of the NF-κB is detected in SG and PBMCs of pSS patients and that phosphorylation of NF-κB is linked to increased infiltration-related disease severity in SG (60, 61). By blocking NF-κB, IκB-α downregulates inflammatory cytokines (44, 62). Peng et al. found that defective IκBα-mediated feedback regulation activates the NF-κB pathway in mouse T cells, promoting the production of inflammatory factors and triggering pSS (62). Epithelial-stromal interaction showed elevated expression in B lymphocytes from pSS patients, activating TLR9 signaling, which promotes p65 phosphorylation and IκBα degradation, activates the NF-κB signaling pathway, and causes aberrant B cell activation (63). TNFAIP3 binds to TNIP1 and blocks the NF-κB pathway (64, 65). There is an association between TNFAIP3 and pSS (65–67). TNFAIP3 deficiency has been found to participate in pSS-associated lymphomagenesis (68, 69), the mechanism of which is related to the germline gene abnormalities with subtle effects on NF-κB activation under continuous stimulation by autoimmune B cells (68, 69). TNIP1 polymorphisms in the NF-κB pathway are linked to antibody positivity in pSS patients (70).

**Table 2:** The activation characteristics and effects of STING in different organs in Sjogren’s syndrome.

Affected organs	STING activation trigger	Activated molecules and cellular effects	Research model	References
Salivary gland	DMXAA, mtDNA, calcium ion concentration	Increased expression of pro-inflammatory factors (IFN-α, IFN-β, IL-6, TNF-α), infiltration of inflammatory cells, production of autoantibodies, high expression of positive regulatory factors such as TRIM38 and TRIM56, and downregulation of RNF26	female C57BL/6 mice, pSS patients’ SG, and primary SG cells	(22, 40, 76–80)
Lacrimal gland	Genomic DNA	Activation of the NF-κB pathway, upregulation of IFN-β expression, promotion of IL-1β secretion, activation of the AIM2 inflammasome, promotion of IL-1β and IL-18 release, induction of cell pyroptosis, inhibition of myoepithelial cell contractile function	NOD mice	(55, 81, 82)
Lung	DMXAA	The expression of pro-inflammatory genes (Ifnb1, Mx1, Il6, Tnf, Ifng, etc.) increases, the number of type 1 innate lymphocytes (ILC1) increases, CD3 ^+^ T cells and antigen-presenting cells aggregate, the number of lymphatic endothelial cells increases, and lymphatic remodeling occurs	female C57BL/6 mice	(83, 84)

However, direct human evidence regarding the activation of STING in SS is still limited, and the mechanism by which its activation primarily affects salivary and lacrimal glands is not yet clear. SS patients have severe ultrastructural changes in the mitochondria of SGEC (20), indicating that SG has unique anatomical structures and functions, which may make them more susceptible to STING pathway activation. Increased IFN-induced gene expression profile was detected in the saliva of SS patients (71). Single nucleotide polymorphism (SNP)-specific sequencing revealed an increase in GTF2I expression in SG at the risk allele of GTF2I SNPs, leading to activation of the NF-κB pathway (72). In addition, the demethylation of SGEC in SS patients was associated with a 7-fold decrease in DNA methyltransferase (DNMT) 1 activity and a 2-fold increase in Gadd45 alpha expression (73). This suggests that genetic background and epigenetic modifications may play important roles in the activation of the STING pathway in SG. Epstein-Barr virus was frequently detected in SG of SS patients (74, 75). This suggests that environmental factors, such as viral infections, may selectively affect salivary and lacrimal glands, leading to intracellular DNA accumulation and activation of the cGAS-STING pathway.

### 4.1 STING activation in SG

Research demonstrates that IFN-α activates the JAK1/STAT1/2 signaling cascade in SGEC, which triggers the release of CXCL13, BAFF, and CXCL10, potentiates lymphocyte stimulation, and facilitates the migration of lymphocytes to SGEC (85). IFN-γ induces the secretion of BAFF by SGEC, which in turn drives the secretion of characteristic autoantibodies (notably IgG and SSA/Ro) (86). Within predominant cell lineages affected by pSS, IFN-α2b activates pSTAT1 Y701 and inhibits the phosphorylation of STAT5 (Y694) and STAT3 (Y705). IFN-γ induces an increased expression of pSTAT1(Y701) in B cells, monocytes, and conventional dendritic cells of patients, which in turn enhances the stimulation state of CD8+ and CD4+ T memory cell subsets and enhances the activation of type I and II T cells and enhances response to type I and II IFN, which was more pronounced in SSA+ patients (87, 88). In SS patients with SG, IFN-α induces high TLR7 expression in pDC, which in turn drives IFN-α synthesis while sustaining inflammatory response (89). Type I IFN induces hsa-miR-145-5p expression while increasing TLR4 and MUC1 transcript levels. IFN-α or IFN-β downregulates hsa-miR-145-5p expression in a manner that dependently perpetuates SG inflammation in SS patients (90). The expression of hsa-miR-145-5p was found to be negatively linked to type I IFN scores, IFN-β levels in the SG of SS patients, mRNA levels of MUC1 and TLR4, as well as Ro/La autoantibody titers and lesion scores of the patients (90). SS patients demonstrate upregulated ISG15, caspase-1, and IL-18 in SG tissues and serum (91). Increased secretion of GSDMD and caspase-1 in the SG epithelium of SS patients has been linked to expression of the signature type I IFN gene. This phenomenon is in relation to upregulation of the hallmark genes of type I IFN (91). *In vitro* studies demonstrated that IFN-α/β induces caspase-1-dependent cell death through GSDMD cleavage following inflammasome stimulation (91). The above results imply that a role for IFN-α/β may promote the inflammatory body-associated pyroptosis process in SGECs from SS patients.

Dimethylxanthenone-4-acetic acid (DMXAA) is similar to mouse STING in an endogenous agonist manner, binding, and promoting the secretion of IFN–β (92). After injecting DMXAA into female C57BL/6 mice, the expression of Il6 (115X), Tnfa (20X), Ifng (23X), Ifnb1 (6X), and Il12p40 (25X) in the SG was significantly upregulated. The expression of IFN-α, IFN-β, IL-6, and TNF-α in the mouse salivary glands increased, inducing inflammatory cells to infiltrate the salivary glands, produce autoantibodies, trigger salivary gland inflammation, inhibit salivary gland function, and promote the onset of SS (25). The expression of STING in the SG of female C57BL/6 mice is mainly observed in ductal and stromal cells (25). *In vitro* studies have found that DMXAA triggers the STING pathways and induces IFN-β production in primary SG cells (25). pSS SG monocytes are hyper-responsive to STING stimulation, as evidenced by an increase in the count of monocytes producing IFN-α. In SG of pSS patients, expression of STING in monocytes and epithelial cells of the infiltrated ducts (40). Production of SENP7, TRIM38, USP18, and TRIM56, which positively regulate STING in monocytes of pSS patients, was markedly elevated compared to healthy individuals, whereas expression of the bifunctional STING regulator RNF26 was downregulated (40). In SS patients, the salivary acinar and ductal SGEC are in an ER stress-activated state (93). TRIM29 is a multifunctional protein belonging to the TRIM family of E3 ubiquitin ligases, which targets STING degradation through protein ubiquitination (94). TRIM29 directly interacts with PERK and induces its stability through protein SUMOylation, thereby promoting PERK-mediated ER stress immune response (95). Upregulation of TRIM29 increases the level of intracellular ROS, which regulates TBK1 through oxidation and inhibits its function, thereby reducing the production of type I IFN (95). In SG of pSS patients, PERK-mediated ER stress response and TRIM29 are activated (96, 97). TRIM29 may regulate the type I IFN signaling pathway by promoting PERK-mediated ER stress, thereby affecting the localization and function of STING; on the other hand, TRIM29 may weaken the negative regulation of intracellular ROS and STING pathways, leading to the production of type I IFN. In-depth study of the specific mechanisms by which TRIM29 regulates these two pathways is expected to provide new targets and strategies for the treatment of SS.

SG secretion is neuromodulated (such as parasympathetic release of acetylcholine). Acetylcholine binds to receptors and activates phospholipase C via G proteins to produce IP3. IP3 binds to receptors on the ER and facilitates Ca^2+^ emission from the ER into the intracellular space, resulting in cyclic Ca^2+^ concentration changes. Elevated intracellular Ca^2+^ concentration triggers subsequent secretory processes, such as facilitating water and electrolyte transport in adenohypophysis (98, 99). In the SG of mice given DMXAA, the STING accessory complex was aberrantly activated and Ca^2+^ signaling was dysregulated, but the level of intracellular Ca^2+^ signaling in the SG cells was elevated after neural stimulation, and salivary secretion was reduced. Under muscarinic stimulation, the decrease in the function of the Ca^2+^-initiated Cl^-^ pathway TMEM16a was accompanied by the disruption of the spatial co-localization relationship between IP3R and TMEM16a (76). Therefore, the defective function of Cl^-^ secretion-related pro-secretin activation may be a key pathologic mechanism of reduced SG secretion in early SS. In addition, this study found that mitochondrial dysfunction and impaired stress response were present in SG alveolar cells of DMXAA-treated SS mice (76). Ca^2+^ finely regulates cellular metabolic processes through the ER-mitochondrial signaling axis, and when Ca^2+^ signaling is dysregulated, Ca^2+^ signaling between the ER and the mitochondria is dysregulated. Ca^2+^ transport balance between the ER and mitochondria is disrupted, which in turn triggers mitochondrial dysfunction (77, 78). It has been found that in SS patients, mitochondria of SG cells show severe ultrastructural alterations (20). When mitochondria expel mtDNA into the cytosol, it can specifically engage cGAS, thereby stimulating the STING-IRF3-mediated pathway, which leads to the overexpression of IFN-stimulated genes, inducing a sustained inflammatory response and ultimately participating within the disease mechanisms of autoimmunity (79, 80). It was found that within SG tissue from SS individuals, mtDNA binding to cGAS activated the STING cascade, which further triggered the NF-κB and type I IFN immune cascade, resulting in the upregulation of transcript abundance of inflammatory mediators, exemplified by IFN-α, IL-8, IL-6, IFN-β, and TNF-α (22). This suggests that damaged mitochondria cause dysregulation of intracellular Ca^2+^ signaling through cGAS-mediated STING cascade stimulation and that aberrant calcium regulation can further exacerbate mitochondrial damage, perpetuating inflammation and forming a vicious cycle. Therefore, targeted inhibition of the cGAS-STING cascade represents a promising therapeutic target in SS. In addition, relevant studies have shown that alterations in intracellular Ca^2+^ levels markedly affect the stimulation process of the STING pathway. Either depletion of intracellular calcium ions by the BAPTA-AM chelator or induced elevation of calcium ion concentration by ionomycin can effectively inhibit the ER-to-Golgi trafficking of STING that subsequently suppresses IFN-β production (100). This phenomenon suggests that STING cascade induction may depend on the precise mediation of intracellular calcium ion concentration, suggesting that the regulation of calcium homeostasis has an important potential value in intervening during pathological STING pathway hyperactivation. It is noteworthy that Yeo-Jun Yoon et al. successfully constructed long-term culturable SG organoids in mice and humans by optimizing the culture system (99). The organoids highly reproduced the structural characteristics of SG tissues and the heterogeneity of gland-specific secretory functions at the phenotypic and functional levels; meanwhile, the culture system was also able to produce tumor-like models with tumor-specific biological characteristics for benign and malignant SG tumors (99). This technological breakthrough provides an ideal *in vitro* platform for comprehensive research on STING-mediated pathogenesis in SG diseases, including SS, and for evaluating intervention strategies targeting calcium homeostasis or the STING pathway.

### 4.2 STING activation in the lacrimal gland

Genomic DNA (gDNA) has been shown to elicit a STING-dependent signaling cascade in NOD mouse lacrimal glands. Subsequent to STING initiation, it will secondarily trigger the NF-κB pathway, enhance IFN-β expression, and then promote the secretion of IL-1β (55). In lacrimal gland myoepithelial cells, homologous gDNA, in addition to triggering the STING pathway, also stimulates Absent in Melanoma 2 (AIM2) inflammatory vesicles, which inhibit the contractile function of myoepithelial cells, upregulate pro-inflammatory mediator secretion, induce apoptosis, and promote inflammatory responses, and thus may drive pSS pathogenesis (55). This study further demonstrated that the inhibition of contractile function of myoepithelial cells did not correlate with intracellular Ca^2+^ levels. *In vitro* experiments have shown that suppressors aimed at the STING protein or AIM2 inflammasome alleviate the inflammatory response (55). AIM2, as an intracellular DNA receptor, is a member of the group of IFN-induced HIN-200 proteins, whose structure consists of a hematopoietic expression, an IFN-inducible property, a HIN structural domain, and a PYD composition (81). The HIN domain activates AIM2 by non-selectively binding to exogenous and endogenous dsDNA. After AIM2 activation, the adaptor protein ASC (speck-like protein associated with apoptosis) is recruited via the PYD domain for further recruitment of procaspase-1, which forms the inflammasome complex. Catalytically active caspase-1 cuts IL-1β and IL-18 precursors into their mature forms, driving inflammatory cell death (81, 82). gDNA can activate the assembly process and biological functions of the AIM2 inflammasome and cause myoepithelial cell secretion of IL-1β and IL-18. Among other things, the stimulatory effect of gDNA on IL-18 secretion is synergistically mediated by post-translational alterations and protein synthesis elevation, whereas modulation of IL-1β production is only a modification effect at the post-translational level (55).

### 4.3 STING activation in the lungs

Early features of lung invasion in SS patients are inflammatory cell infiltrates in the peribronchial region (41). In the later phases of the disease, fibrosis shifts in interstitial pulmonary disease are the principal causes, in which lymphatic remodeling is in the process of pulmonary fibrosis, playing a key role (101). After delivering DMXAA subcutaneously to female C57BL/6 mice, the STING cascade was induced, which resulted in a crucial induction in the transcriptional levels of inflammatory mediators, notably Ifng, Mx1, Il6, Ifnb1, and Tnf in pulmonary tissue; a tremendous increase in the number of Intrinsic Lymphocyte Type 1 (ILC1) cells in the lung tissues, lymphocyte infiltration of the lungs (mainly aggregated in the peribronchiolar region), and inflammatory foci consisting mainly of CD3+ T cells and MHC II+ antigen-presenting cells. In addition, activated STING induced a marked proliferation of lymphatic vessel endothelial cells in the lungs, a phenomenon that suggests the presence of lymphatic remodeling, a prospective pre-presentation of fibrotic changes in the lung (83). The inflammatory response in SS mucosal tissues is significantly driven by epithelial cells, where STING activation in respiratory epithelium may intensify pro-inflammatory signaling. In contrast, STING protein expression was detectable in bronchial epithelial cells and alveolar wall cells of female C57BL/6 mice (83). STING-dependent IFN production involves cells of both hematopoietic origin and non-hematopoietic lineages, but the intracellular inflammatory response within an organ relies on the expression of STING on hematopoietic cells, which triggers elevated expression of early inflammatory mediators contributing to the migration of ILC1 to pulmonary tissue and the formation of inflammatory foci in the lungs (83). There is a significant correlation between the lip-gland biopsy lesion scores and oral dryness in pSS with the pulmonary manifestations, in which the focus score and oral dryness could indicate concurrent lymphoid infiltration in pSS lungs (84). The above study indicates STING pathway activation could contribute to the co-occurring salivary and pulmonary pathology in SS patients (83).

## 5 STING pathway-targeted interventions

With recent studies revealing that the cGAS-STING cascade is central to autoimmune diseases, the emergence of inhibitors targeting this pathway has become a research hotspot. Currently, the development of pathway-specific inhibitors has achieved milestones involving peptides, small molecule compounds, and inhibition based on covalent and non-covalent interactions mode (30, 102, 103). For example, ISD017, which ameliorated the illness development in a lupus mouse model and did not show significant effects in terms of cytotoxicity, inhibited the ER-to-Golgi translocation of STING in a STIM1-dependent manner, which, in turn, effectively blocked the STING-mediated signaling pathway activation (104). In light of STING’s critical function in SS pathogenesis, inhibiting STING has been observed to substantially dampen the inflammatory cascade in experimental SS mice (**Table 3**) (25).

**Table 3:** Therapeutic targets and intervention effects of the STING pathway in Sjogren’s syndrome.

Category of intervention strategies	Pathway	Mechanism of action	Related drugs	Research phase	Core effect	References
Autophagy regulation	Autophagy-STING interaction	Autophagy clears damaged mitochondria and leaked mtDNA, reducing cGAS activation substrates. Autophagy directly degrades STING-related complexes, inhibiting pathway activation.	Rapamycin	Animal experiments (NOD mice)	Relieve inflammation of salivary/lacrimal glands, increase secretion function, and inhibit activation of the cGAS-STING pathway.	(105–111)
Ca^2+^ steady-state regulation	STIM1-STING pathway	STIM1 anchors STING to the ER, preventing its transport to the Golgi apparatus. Intestinal microbiota (such as L. acidophilus) inhibits STING activation by promoting STIM1 expression.	Probiotics (Lactobacillus acidophilus)	Animal experiments (NOD mice); small-sample clinical exploration	Reduce the production of type I IFN, inhibit lymphocyte infiltration, and improve symptoms of dry mouth and eyes.	(104, 112–118)
Directly blocking the STING pathway	cGAS-STING pathway	Hydroxychloroquine binds to the DNA binding site of cGAS, blocking its interaction with dsDNA. Inhibits the secretion of IFN and pro-inflammatory cytokines downstream of STING.	Hydroxychloroquine	Widely used in clinical practice	Reducing IFN score and related gene expression, improving inflammatory indicators, and effectively treating symptoms in some patients.	(13, 119–124)

### 5.1 Autophagy and STING pathway

Autophagy, the cellular mechanism for eliminating impaired or superfluous organelles, is crucial for preserving intracellular balance and may also be a crucial mechanism in the modulation of organelle and protein homeostasis in SG vesicle cells (125). When autophagy is deficient, it triggers the STING cascade and inflammatory polarization (105, 106), while lowering chromatin fragment accumulation in the cytoplasm (107). A recent study found that autophagy reduced triggering of the cGAS/STING pathway in NOD mouse SMG (108). Therefore, inhibiting cGAS-STING pathway activation through autophagy modulation offers novel therapeutic potential for SS. This suggests that the crosstalk between autophagy and STING signaling could represent a critical pathogenic mechanism in SS. The mtDNA-packaging protein TFAM (mitochondrial transcription factor A) can interact with autophagy protein LC3 via the autolysosomal pathway to remove leaked mtDNA and reduce the substrate for cGAS activation, which in turn inhibits excessive STING signaling induction and controls inflammation (109).

Rapamycin, an immunomodulator, topical application of rapamycin inhibits lacrimal gland lymphocyte infiltration, lessens corneal fluorescein staining, enhances tear secretion, alleviates inflammatory damage in the lacrimal gland, and suppresses Cathepsin S proteolytic activity in both tear fluid and lacrimal gland lysates and decreases Catepsin S enzymatic function in lacrimal secretions and tissue homogenates from NOD mice, as well as regulating the transcriptional profile of key SS-related mediators (Akt3, IL-12a, IFN-γ, MHC-II, and TNF-α) (110). Rapamycin inhibits the activation of the cGAS-STING pathway by promoting autophagy, which in turn alleviates SG pathological changes in SS model mice (108). In addition, rapamycin microspheres ameliorated corneal histological damage, mitigated corneal endothelial injury, improved the dry eye symptoms, and stimulated tear output in NOD mice (111). There are fewer clinical trial studies on rapamycin for the treatment of SS. Therefore, rapamycin may control disease progression by regulating autophagy, indicating a promising treatment avenue for SS. In the future, clinical trials are required to evaluate its effectiveness and safety in different groups of patients with SS.

### 5.2 STIM1 and STING pathways

STIM1 acts as a calcium ion receptor on the ER, anchoring STING to the ER and preventing its translocation, thereby blocking the STING pathway (104, 112, 113). When STIM1 is absent, the STING signaling pathway is over-activated, driving upregulation of type I IFN (113). Research has shown that intestinal microbial diversity and abundance decline in SS patients, and the proportion of pro-inflammatory opportunistic pathogens increases, thereby inducing pro-inflammatory cytokine generation, inhibiting the release of anti-inflammatory factors, and disrupting intestinal barrier function, thereby exacerbating the occurrence of inflammatory reactions (114). Gut microbiota dysbiosis is present in juvenile and aged NOD mice, and Lactobacillaceae and Lactobacillus numbers are reduced in older and younger NOD mice (115). L. acidophilus downregulates the secretion of inflammatory cytokines (IL-6, IL-17 and TNF-α), inhibits lymphocyte infiltration, improves saliva production, and alleviates SG inflammation, thereby alleviating SS symptoms (115). The mechanism includes, on the one hand, L. acidophilus inhibiting the STIM1-STING pathway and diminishing type I IFN synthesis; on the other hand, Lactobacillus acidophilus inhibiting lymphocyte recruitment to SG by promoting the production of propionic acid, promoting the expression of STIM1, reducing the production of STING, and inhibiting type I IFN overproduction to alleviate SS symptoms. Production to alleviate SS symptoms (115). At present, research on treating SS by regulating gut microbiota (e.g., gut microbiota transplantation or probiotic therapy) is still in the exploratory stage, but existing studies have shown the potential application of gut microbiota in SS (116). For example, a nonrandomized clinical trial found that gut microbiota transplantation can effectively improve dry eye symptoms in immune-mediated dry eyes (117). Another randomized controlled trial showed that versus placebo-treated controls, the probiotic capsule group (containing Lactobacillus acidophilus, Bifidobacterium bifidum, etc.) can reduce the Candida burden in the oropharyngeal mucosa of SS subjects (118). In summary, the strategy of regulating gut microbiota to treat SS may provide new ideas for developing personalized treatment plans.

### 5.3 Hydroxychloroquine and the STING pathway

Hydroxychloroquine (HCQ) is widely used as an immunomodulator for treating multiple autoimmune conditions. The clinical use of HCQ in the treatment of SS ranges from 25% to 50.6% (119–121). HCQ improves gut microbiota by regulating IFN, chemokines, and BAFF levels, thereby exerting immune-modulating and inflammation-suppressing activity, but its clinical efficacy in SS is still controversial (119). Meta-analysis/systematic review data revealed non-significant treatment effects for HCQ *vs*. placebo in treating SS-associated xerostomia and xerophthalmia; the efficacy of HCQ in treating SS-related fatigue was less effective than placebo but superior to placebo in improving pSS-related pain and reducing erythrocyte sedimentation rate (122). A 2021 pooled analysis demonstrated that the HCQ group was significantly better than the non-HCQ group in improving resting salivary output, immunoglobulin profiles (IgM/IgA), and acute-phase markers (CRP/ESR) (13). Jie An et al. predicted that HCQ interacts with the DNA of cGAS by computerized drug library screening analysis (123). Evidence from *in vitro* systems indicates (123) that HCQ inhibits IFN-β production in a dose-dependent manner by blocking cGAS DNA interactions in human monocyte lines transfected with dsDNA, with a maximum inhibitory concentration of 25 μM. A 2022 clinical trial showed that, versus placebo controls, HCQ can reduce IFN scores and gene expression of key IFN-I-inducing DDX58, IFIH1, IFI16, ZBP1, and IFI16, and the decrease in ESR, IgG, and IgM levels is not related to the patient’s IFN activation status (124). Advancing the evidence base requires multicenter randomized trials coupled with long-term effectiveness appraisals.

## 6 Summary and outlook

SS, as an autoimmune disease mainly characterized by chronic inflammation of exocrine glands, is closely related to its pathogenesis and abnormal immune system responses. The STING pathway, as a key hub for intracellular nucleic acid sensing, plays a “bridge-like” regulatory role. This article systematically reviews the complete molecular chain of the STING pathway involved in the pathogenesis of SS: The abnormal release of endogenous DNA (cf-DNA, mtDNA) provides a core substrate for STING activation; nucleic acid sensors such as cGAS, IFI16, DDX41, and ZBP1 activate STING through specific mechanisms such as cGAMP mediation and direct protein interaction. Abnormal activation of STING further triggers tissue-specific pathological damage in salivary glands, lacrimal glands, and lungs, and amplifies inflammatory effects through downstream type I interferon pathways and NF-κB pathways. In addition, the expression imbalance of regulatory factors such as TRIM21 and TRIM29 in the TRIM family (such as TRIM21 functional defects leading to DDX41 accumulation) further exacerbates the overactivation of the STING pathway, forming a vicious cycle of “injury-inflammation-reinjury”.

At present, research on treating SS by inhibiting the STING pathway is still in the exploratory stage. Previous studies have found that autophagy regulators (such as rapamycin) alleviate glandular inflammation by clearing mtDNA and degrading STING complexes; STIM1-mediated Ca^2+^ homeostasis regulation (including probiotic-induced STIM1 expression) can inhibit STING transport; Hydroxychloroquine is activated by blocking the cGAS DNA binding inhibition pathway, and some strategies have shown potential in animal experiments (such as NOD mice) or small-sample clinical exploration. However, existing research still has clear limitations: Firstly, clinical evidence of direct activation of STING in human SS (such as the correlation between STING phosphorylation levels in glandular tissue, cGAMP concentration, and disease activity) is still lacking; Secondly, the mechanism of tissue-specific activation of the STING pathway (such as the molecular basis of high sensitivity in salivary/lacrimal glands) has not been fully elucidated; Thirdly, there are bottlenecks in the clinical translation of treatment strategies, such as the lack of SS-related clinical trials for rapamycin, controversy over the efficacy of hydroxychloroquine and the need for long-term safety data, and the exploration of emerging strategies for regulating gut microbiota. Future research can focus on breakthroughs in the following directions: Firstly, deepening mechanism research, focusing on key nodes of the STING pathway regulatory network (such as TRIM29’s bidirectional regulation of PERK-ER stress and STING, and Ca^2+^-mitochondrial STING interaction), and combining *in vitro* models such as salivary gland organs to analyze tissue-specific mechanisms. Secondly, develop specific biomarkers such as cf-DNA subtypes, STING downstream effector molecules (IFN-β, IL-6), or pathway regulatory factors (such as PARP9) for early diagnosis and disease stratification of SS. Thirdly, optimize treatment strategies by promoting the development of tissue-targeted agents (such as salivary gland/lacrimal gland-specific STING antagonists) and exploring combination schemes (such as the combination of autophagy regulators and STING antagonists) to balance efficacy and immune homeostasis. In addition, the successful experience of STING agonists in cancer immunotherapy (126) also provides ideas for the innovation of SS treatment strategies. Given that STING protein is widely expressed in various types of cells so as to improve drug efficacy, augment treatment effectiveness, enhance targeting specificity, and reduce adverse reactions, STING inhibitors can be prepared into antibody drug conjugates (127, 128) or delivery systems such as nanoparticles and liposomes (129–131) to enable precise targeted drug delivery to diseased tissues, thereby effectively enhancing the therapeutic efficiency of SS patients. However, pharmacological studies related to STING inhibitors, particularly their toxicity and potential side effects, are still lacking. Therefore, whether long-term inhibition of the STING pathway increases infections, tumors, or other adverse reactions also needs to be further evaluated through rigorous clinical studies. It is worth noting that SS patients have individual heterogeneity in clinical manifestations and pathological mechanisms, which requires clinical treatment to break through traditional models and develop precise and customized treatment strategies based on individual patient characteristics. With the continuous deepening of research and steady progress of clinical trials, the STING pathway emerges as a promising breakthrough point in the treatment of SS, bringing more treatment options for patients.
